# Redo extended thoracic aortic replacement from aortic root to descending aorta via anterolateral thoracotomy with partial sternotomy for graft infection

**DOI:** 10.1186/s44215-024-00144-8

**Published:** 2024-02-27

**Authors:** Tasuku Okada, Yojiro Koda, Katsuhiro Yamanaka, Kenji Okada

**Affiliations:** Division of Cardiovascular Surgery, Department of Surgery, Kobe University Graduate School of Medicine, 7-5-2 Kusunoki-cho, chuo-ku, Kobe, Hyogo 650-0017 Japan

**Keywords:** Graft infection, Anterolateral thoracotomy with partial sternotomy, Redo operation, Bio-Bentall procedure

## Abstract

A 59-year-old male underwent Bio-Bentall + total arch replacement with a frozen elephant trunk for acute type A aortic dissection before at another hospital. He was diagnosed as mediastinitis and previous graft infection, followed by wound closure with omental flap installation. However, the recurrent graft infection from the aortic root to the FET in the descending aorta was diagnosed by 18-fluorodeoxyglucose positron emission tomography. Redo modified Bio-Bentall procedure, total arch replacement, and descending aortic replacement for previous graft infection using anterolateral thoracotomy with partial sternotomy was successfully performed. Anterolateral thoracotomy with partial sternotomy provided not only the excellent exposure from the aortic root to the descending aorta but also sure myocardial protection with antegrade and selective delivery of cold crystalloid cardioplegia and stable brain protection with antegrade selective cerebral perfusion. The patient is doing well without recurrent of infection after 2 years of the operation.

## Background

Redo open thoracic aortic repair is increasingly performed for extensive stent/prosthetic graft infection. Extensive replacement predisposes to in hospital mortality [1]. Selection of subsequent surgical approach is important for aortic replacement from aortic root to descending aorta. In this case report, we successfully performed redo modified Bio-Bentall procedure, total arch replacement (TAR), and descending aortic replacement for previous graft infection using anterolateral thoracotomy with partial sternotomy (ALPS) [2].

## Case presentation

**Fig. 1:**
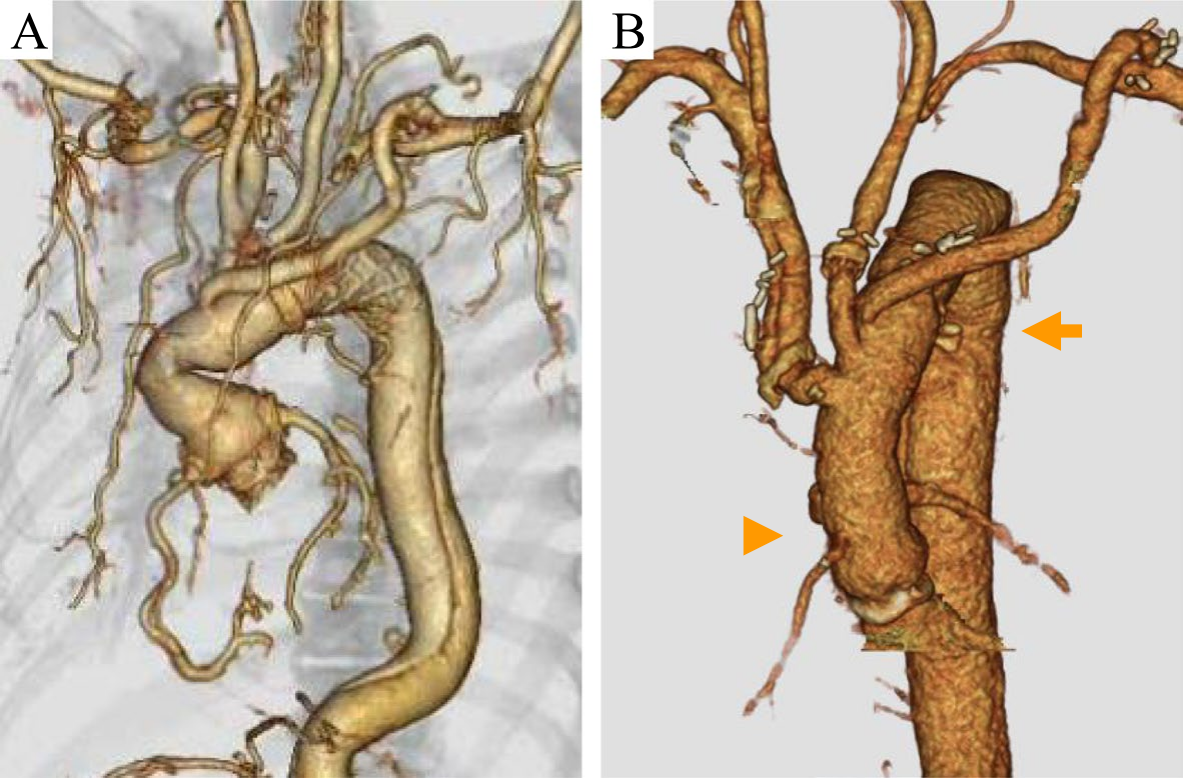
**A** Preoperative 3D CT showing primary extensive aortic arch replacement with FET, including the aortic root replacement using Bio-Bentall procedure. **B** Postoperative 3D CT. Arrowheads show the proximal composite graft and arrow shows the level of distal anastomosis

A 59-year-old male presented with high-grade fever. He underwent Bio-Bentall (21 mm, INSPIRIS RESILIA, Edwards Lifesciences LLC, Irvine, CA, USA, 28 mm, Gelweave Valsalva graft, Terumo Vascutek, Tokyo, Japan) + TAR (24 mm, J-Graft, Japan Lifeline, Tokyo, Japan) with a frozen elephant trunk (FET, 29 × 60 mm, J Graft FROZENIX, Japan Lifeline, Tokyo, Japan) for acute type A aortic dissection 2 years previously at another hospital. He was diagnosed with mediastinitis and previous graft infection. He underwent vacuum assisted closure, followed by wound closure with omental flap installation (Fig. 1A).

**Fig. 2:**
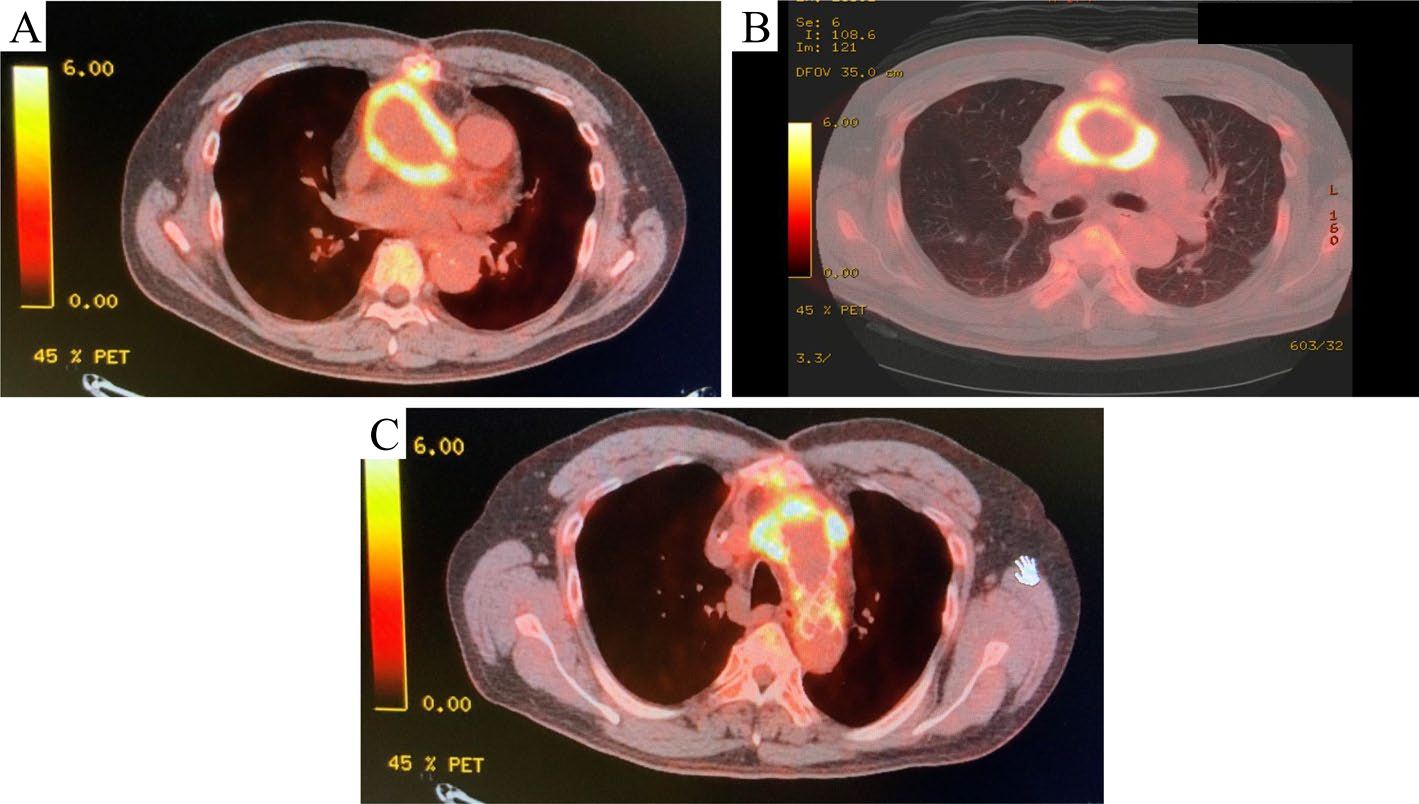
18-Fluorodeoxyglucose positron emission tomography (PET) showed significantly elevated SUV value. **A** Level of the aortic root, **B** ascending aorta, **C** FET

After 10 months later, the patient developed fever and the blood culture revealed growth of *Achromobacter xylosoxidans*. Although contrast-enhanced computed tomography showed little air or fluid collection, 18-fluorodeoxyglucose positron emission tomography showed significantly elevated standardized uptake value (SUV) [3] in the prosthetic grafts except the distal part of prosthetic arch vessels (Fig. 2A, B, C). Redo open surgery using ALPS was performed for extensive replacement.

**Fig. 3:**
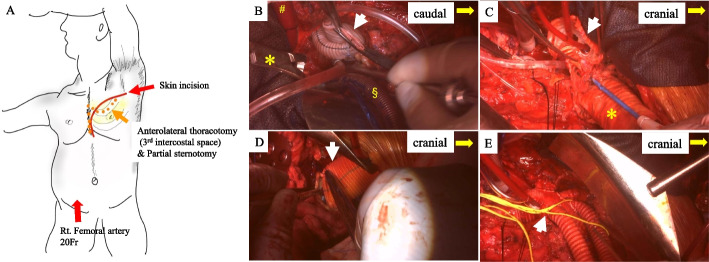
**A** Surgical schema showing the patient’s position and incisions for ALPS. **B** White arrow shows the rifampicin-bonded aortic root composite graft with Svensson method. * Aortic cross clamp on the previous prosthetic graft, # aortic return cannula in the previous prosthetic graft, § a venous drainage cannula in the SVC. **C** White arrow shows that selective balloon tipped cannulae were inserted into BCA and LCCA. * FET. **D** White arrow shows that a prosthetic tube graft was anastomosed to the descending aorta using the open distal method. **E** White arrow shows that the aortic arch was reconstructed using a new 4-branched prosthetic graft*

**Fig. 4:**
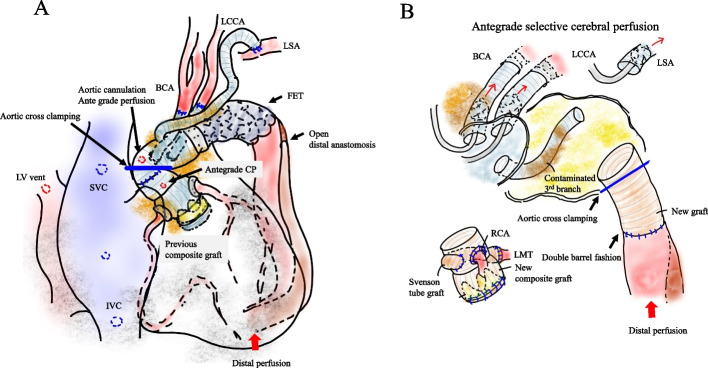
**A** Cardiopulmonary bypass was established by bicaval drainage and ascending aortic prosthetic graft return. The left ventricle was vented through the right upper pulmonary vein. After the cross clamping on the ascending aortic prosthetic graft, antegrade cardioplegia was delivered. BCA brachiocephalic artery, LCCA left common carotid artery, LSA left subclavian artery, FET frozen elephant trunk, CP cardioplegia. **B** Antegrade selective cerebral perfusion was securely established through a fine visual field

**Fig. 5:**
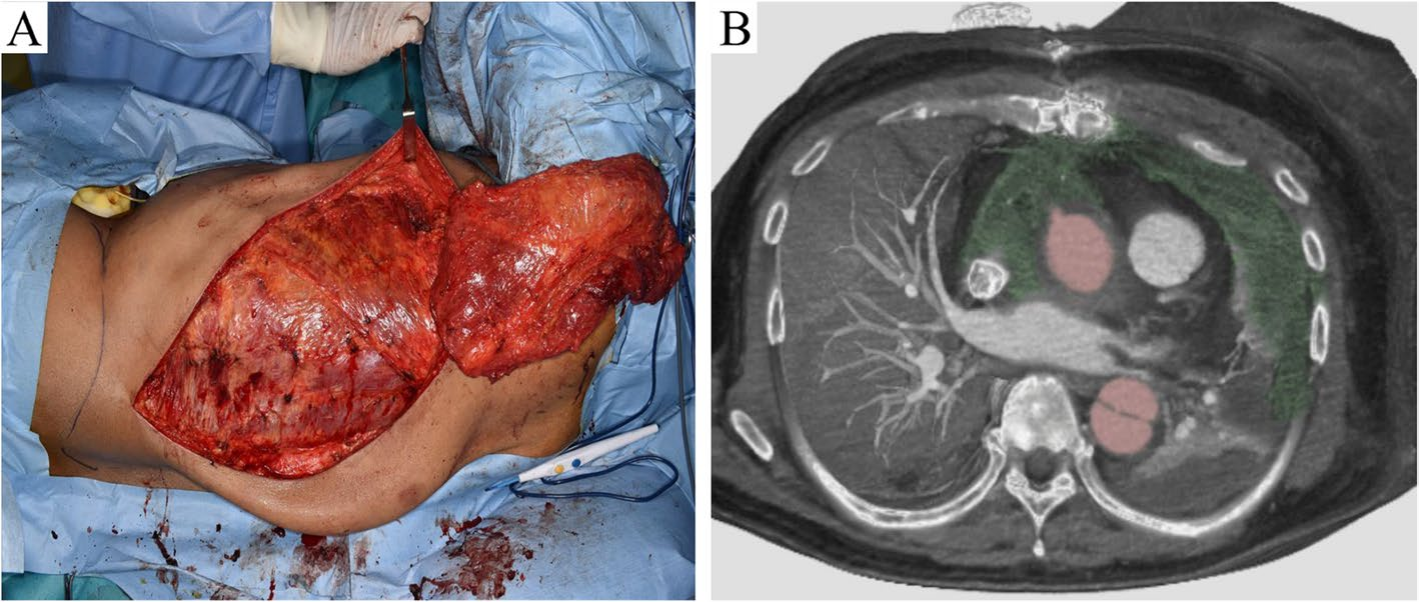
Latissimus dorsi flap grafting. **A** Latissimus dorsi flap was prepared 8 days after operation. **B** Postoperative CT findings after implantation of the latissimus dorsi flap (green area)

In the right semi-recumbent position with the left arm elevated towards the head (Fig. 3A), the previous omental flap was dissected. The infected prosthetic grafts were exposed through anterolateral third thoracotomy and lower partial sternotomy. After systemic heparinization, cardiopulmonary bypass (CPB) was established using bicaval drainage and aortic/arterial return into the ascending graft (20 Fr) and right femoral artery (20 Fr). A left ventricle cannula was inserted through the right upper pulmonary vein. The patient was cooled to a rectal temperature of 33 °C. The ascending aortic graft was cross-clamped and antegrade cardioplegia was delivered to obtain cardiac arrest (Fig. 4A). Since Valsalva graft infection was confirmed by both macroscopic and intraoperative microscopic examinations, redo Bio-Bentall was first performed using a 21-mm bioprosthetic valve (INSPIRIS RESILIA, Edwards Lifesciences LLC, Irvine, USA) and rifampicin bonded 24-mm Valsalva graft (Gelweave Valsalva graft, Terumo Vascutek, Tokyo, Japan). Left coronary artery was reconstructed using the modified Cabrol method (Svensson method) using 10-mm tube graft (Gelweave, Terumo Vascutek, Tokyo, Japan), because of the fragile tissue and insufficient mobilization (Fig. 3B). During the aortic root procedure, rectal and tympanic temperatures were maintained at 30 °C and 23 °C, respectively, to achieve hypothermic circulatory arrest [4]. The aortic arch graft was opened to remove FET, and 15- and 12-Fr selective balloon tipped cannulae (Sumitomo Bakelite, Akita Japan) were inserted to the brachiocephalic artery (BCA) and the left common carotid artery (LCAA) (Fig. 3C). The previous left subclavian artery (LSA) graft was exposed at the level of second intercostal space, followed by 12-Fr cannula insertion (Fig. 4B). Descending aortic replacement was performed via open distal anastomosis using 4–0 polypropylene (Fig. 3D). The distal anastomosis was done by double barrel fashion. The straight graft was clamped, followed by lower body reperfusion and rewarming through right FA perfusion. Then, 4-branched J-Graft (24/11/9/9, Japan Lifeline, Tokyo, Japan) was anastomosed to the aortic composite graft using 4–0 polypropylene. BCA was reconstructed using the first branch of the J-Graft, followed by the myocardial and brain reperfusion through the side branch of the J-Graft. The distal part of the J-Graft was anastomosed to the descending aortic graft using 4–0 polypropylene. LCCA and LSA were reconstructed using second and long third branches of the J-Graft (Fig. 3E). Previous grafts and felt strips were all removed except the very short part of previous prosthetic arch vessels, which showed negative SUV of PET CT scan. After the aggressive debridement of infected tissue, we irrigated the field with copious saline solution. The patient was weaned from CPB without circulatory support. The CPB time, myocardial ischemic time, and the lower body circulation arrest time were 486, 340, and 36 min, respectively. One week later, the latissimus dorsi flap was implanted around the aortic root, ascending aorta, and arch prosthetic graft (Fig. 5A, B). Intravenous antibiotics were used for 6 weeks, followed by oral antibiotics. The patient recovered well and had no infection in the two postoperative years. Postoperative CT scan is presented in Fig. 1B.

## Discussion

Recent thoracic stent grafts enable extensive aortic arch replacement down to the descending aorta. However, infected grafts (e.g., polyester grafts, FET, and stent grafts) for thoracic endovascular aortic repair require open redo replacements from aortic arch to descending aorta. Although the left thoracotomy is the standard approach for extensive replacement [5], it is not suitable for aortic root replacement, such as redo Bio-Bentall procedure for infection. ALPS has been performed previously [2] and has the advantage of median sternotomy to establish standard cannulation for CPB and the left thoracotomy for descending aortic replacement, enabling complex replacement from the aortic root to the mid descending aorta. Both antegrade and retrograde methods can be used for myocardial protection. Antegrade selective cerebral perfusion may be performing using the standard manner reported previously [4].

## Conclusion

ALPS is excellent for extensive and complex thoracic aortic replacement, including aortic root infection, with myocardial and brain protection.

## Data Availability

The datasets used and/or analyzed during the current study are available from the corresponding author on reasonable request.
